# Evidence-Based Decision Making When Designing Environments for Physical Activity: The Role of Public Health

**DOI:** 10.1007/s40279-015-0469-6

**Published:** 2016-02-02

**Authors:** Paul Pilkington, Jane Powell, Adrian Davis

**Affiliations:** Faculty of Health and Applied Sciences, Department of Health and Social Sciences, The University of the West of England, Frenchay Campus, Coldharbour Lane, Bristol, BS16 1QY UK; Strategic Transport Division, Bristol City Council, Brunel House, Bristol, BS1 5UY UK

## Abstract

The important role that the environment plays in health and well-being is widely accepted, as is the impact that the built and natural environment can have on levels of physical activity. As levels of physical activity are a key determinant of health, promoting physical activity through actions to improve the environment is a priority for public health action. The challenge for public health is to ensure that the way the environment is shaped and transformed by a range of professionals, organisations and agencies, maximises health gain in relation to health, including physical activity. This article discusses how the public health profession can and should contribute to generating and disseminating evidence to inform decision-making processes for designing environments to promote physical activity. There are significant challenges to building and applying the evidence base in this area. These include the complex environments in which interventions operate, disciplinary differences in approaches to evidence generation and use, and the fact that public health has little responsibility for environmental change. However, case studies of best practice, presented in the article, offer a snapshot of how challenges can be overcome, to build an accessible evidence base and help to improve the environment for the promotion of physical activity.

## Key Points

**Table:** 

There are significant challenges to promoting an evidence-based approach when designing environments for physical activity, including the complex environments in which interventions operate, and the disciplinary differences in approaches to evidence generation and use.
Public health should work more closely with the range of professions and organisations that have responsibility for designing environments for physical activity, promoting evidence-based approaches through knowledge and skills transfer across professional divides.
Public health needs to improve the quality and scope of the evidence base in the field, developing methodologies and methods to better evaluate environmental interventions aimed at impacting on physical activity, taking into account the complex systems in which those interventions operate.

## Introduction

The important role that the environment plays in health and well-being is widely accepted, as is the impact that the built and natural environment can have on levels of physical activity [1, 2]. As physical activity is a key determinant of health, promoting physical activity through actions to improve the environment is a priority for public health action. The challenge for public health is to ensure that the way the environment is shaped and transformed by a range of professionals, organisations and agencies, maximises health gain in relation to health, including physical activity. This article discusses how the public health profession can and should contribute to generating and disseminating evidence to inform decision-making processes for designing environments to promote physical activity. It is both a rallying call for public health professionals to engage more effectively with this area, and also aims to raise awareness among a wider constituency (including physical activity specialists) of the importance of taking an evidence-based approach to designing environments for physical activity. Case studies of good practice are used to illustrate the challenges for the effective consideration of evidence.

## Background

Public health is defined as “The science and art of promoting and protecting health and well-being, preventing ill-health and prolonging life through the organised efforts of society” [3]. The public health movement is characterised by its focus on health and well-being of populations, social justice and equity [4]. In the last two decades, there has been an increasing recognition among the public health community of the link between health and well-being and the wider determinants of health, including the built and natural environment [5]. This in turn has necessitated a refocus of public health energies to interventions to improve the physical environment. In many ways, this is a return to the past—many of the most important advances in public health have come through improvement of the physical environment, such as the sanitary reforms of the 18th century or air-quality laws in the mid-1900s [6].

Renewed interest in public health from the 1980s stemmed from the Lalonde Report [7] and the work of McKeown [8], which were catalysts for the re-emergence of public health in the UK and elsewhere. These authors helped raise awareness about the part that social and environmental factors play as determinants of ill health in the emergence of health problems. They highlighted the importance of recognising that health status is influenced by environmental factors beyond as well as under the control of individuals. Health status and health inequalities are influenced more by the circumstances in which people live and by way they live than they are by the provision of health services, though the latter are also important for health and well-being.

This holistic approach is reflected in the definition of health contained in the Constitution of the World Health Organization, where health is defined as “a state of complete physical, mental and social well-being and not merely the absence of disease or infirmity” [9]. In pursuit of this goal, public health is concerned with the determinants of health, the patterns of distribution of those determinants in a population, and also how those determinants and their distribution might be modified ‘through the organised efforts of society’. From a public health perspective, how people live in a city and how healthy and happy they are depends to a considerable extent on their urban environment, their access to employment, to services, to travel and transport, to green space and on the community around them.

As an example, public health has identified “obesogenic” environments (environments that promote obesity) as a key factor in the current obesity epidemic [10]. Poor-quality, unsafe and unappealing environments dissuade people from walking, cycling and engaging in active play. Interventions to improve the environment and promote physical activity and active living include those relating to transport, road safety and safe play [11].

## Physical Inactivity as a Major Public Health Concern

Why does public health have a view on the impact of the obesogenic environment on health and well-being? Humans are designed to be physically active, but modernity has engineered and mechanised physical activity out of everyday living, and time spent sitting has increased as one of the consequences [12]. Leisure time has increased and there are many opportunities and activities to engage in sedentary activities that are screen based, for example, social media, digital media viewed online or by satellite broadcast, terrestrial television, computing and electronic games. Physical activity is a broad term used to describe “any force exerted by skeletal muscle that results in energy expenditure above resting level” [13]. Thus, the term ‘physical activity’ includes any form of human movement including walking, cycling, play, active hobbies or manual occupations as well as structured exercise or sport.

There is overwhelming evidence that regular physical activity has important and wide-ranging health benefits. These range from reduced risk of chronic diseases such as heart disease, type 2 diabetes mellitus, and some cancers, to enhanced function and preservation of function with age. There is also strong emerging evidence that activity delays cognitive decline and is good for brain health as well as having extensive benefits for the rest of the body [14]. Physical activity contributes to wellness, by enabling greater connection with others, with green space, and helping to deal with stress [15–18].

The built, physical, and psychosocial (social and cultural) environments are all important determinants of physical activity [19]. A case-controlled study from Belgium reported that residents in a neighbourhood with high walkability took more steps per day than those in a neighbourhood with low walkability, and they walked more for transport [20]. Further analysis showed that living in a highly walkable neighbourhood was also associated with taking more steps in adults with a preference for passive transport and/or a low intention to walk or cycle. Other studies have also shown that it is the presence of increased opportunities afforded by the built environment to be more physically active that is most influential and not activity-oriented residents choosing to live in certain neighbourhoods [21].

## Evidence-Based Approaches When Designing Environments for Physical Activity

As the impact of the changing environment on population health has become more widely known, the search for solutions has intensified—a quest for ‘what works’. Public health is a multidisciplinary profession, and public health activities demand a wide range of competencies, including expertise in evaluation and promotion of evidence-based policy and practice. Indeed, the focus on evidence is a key skill that public health practitioners can offer in the area of health and environment. Evidence-based public policy is a relatively recent movement. The rise of evidence-based policy and practice was first attributed to medicine, and evidence-based medicine became ‘fashionable coinage’ during the 1990s. In public health, evidence is now understood as a central pillar of public policy decision making, helping to ensure that only interventions that are effective (and cost effective) are promoted. Evidence-based approaches in public health also strengthen advocacy activities, making the case for action to decision makers.

There are important challenges for building an evidence-based approach, to identify how environments can be better designed to more effectively promote physical activity and to then ensure effective approaches are implemented. The first relates to generating evidence for complex interventions that will be delivered within complex systems of the real world. Unlike the environmental health problems of the past, which often required relatively simple solutions, today’s environmental issues, including how the environment impacts on physical activity, are more complex [22]. This can make not only evaluation difficult, but also the transfer of evidence generated from the laboratory conditions of a research study into the real-life conditions of implementation in practice. This issue of internal and external validity remains a challenge therefore even when an intervention in a trial is effective because it may not achieve the same level of effectiveness in a different location (although this important aspect may be less well understood among some practitioners not well versed in methodological issues).

Another challenge for evidence-based approaches is that for some, including those working in areas that impact on environmental determinants of physical activity (such as transport and spatial planners), evidence may be considered more of a second-order consideration once the policy direction has been decided. Thus, the very meaning of evidence is highly contestable. What is accepted as evidence, how much is it valued, and how this differs between academics and practitioners and different professions creates barriers to successful collaboration. This results in a diverse stock of ‘evidence’ drawn on by professions, decision makers and lay people. Moreover, evidence is only one element of a co-production equation that also includes ideological positions, pragmatism and business as usual approaches. Despite the challenges of taking an evidence-based approach, engaging with a range of professions around evidence is crucial, as public health has very little, if any, ability to change the environment to enable and support routine physical activity. Action to improve health and reduce health inequalities through an evidence-based approach therefore requires collaboration with, and sign-up from, a range of other disciplines [4]. In addition, it is becoming clearer, both in the UK and in other Western European countries, that there is a need to develop a shared language around collaboration and the varied meanings of evidence. Pragmatically, it is unrealistic to expect major changes in the short term from transport planning and built environment professionals regarding evidence. There is a clear need for translational research activities to increase simply to provide access to key evidence currently unknown and inaccessible (including because of jargon and paywalls). There may be a role here for more support from funding research councils in meeting this latent demand and thus the impacts of research they fund.

## Evidence into Practice

Currently, the evidence base for public policy and practice does not enable decision makers and practitioners to take action in practice to deliver evidence-based solutions. As we have noted, awareness of evidence-based studies in the field of environmental design is a challenge owing to cultural and professional cultures which, unlike public health (and some other professions such as medicine) become divorced from peer-reviewed literature in the transition from undergraduate and post-graduate students into the workplace. Public health can and should engage with these other professionals to transfer knowledge and skills around evidence-based approaches, demonstrating the value of this approach, not least in providing an audit trail that can better defend the decision-making process.

Then, beyond access to, and the secondary order function of peer-reviewed evidence across urban environment professionals, there are further issues which require consideration. Plausibility and likelihood of success are two different aspects that must be assessed to understand whether complex interventions intended to increase physical activity in the population can deliver health improvements at a population level [23]. A complex intervention to promote physical activity is assessed as plausible when it is demonstrated scientifically through research evidence that it is effective in increasing objectively measured physical activity in a population. Likelihood of success of an intervention in making a population impact is determined by translating scientific evidence into action for a particular context or community. This process relies upon the tacit knowledge and skill of practitioners in understanding need and circumstance, embracing public involvement so interventions with reach and potential for uptake are implemented, and the competence of commissioners and sponsors of physical activity interventions in understanding how to marry evidence of plausibility and likelihood of success. Public health is well placed to facilitate this process, bringing together a range of stakeholders.

The RE-AIM (Reach, Effectiveness, Adoption, Implementation, Maintenance) framework, developed within public health, offers one means to “enhance the quality, speed, and public health impact of efforts to translate research into practice” [24]. It focuses on dimensions for evaluating the potential public health impact of programmes intended for wide-scale implementation and dissemination [25] One example of how the RE-AIM framework has been used in the context of environmental design for physical activity benefits is to evaluate the impact of urban regeneration projects in Belfast on public health, particularly the nature and degree to which urban regeneration impacts upon health-related behavioural change (including physical activity) [25].

The following case studies exemplify some of the issues outlined in this paper, demonstrating the challenges faced and how these were overcome.

## Case Studies

### iConnect Consortium

The iConnect study aimed to measure and evaluate the changes in travel, physical activity and carbon emissions related to Sustrans’ Connect2 programme [26]. This was a UK-wide infrastructure project to create new routes for walking and cycling and transform local travel in more than 80 communities by creating new crossings and bridges to overcome barriers such as busy roads, rivers and railways. The 5-year iConnect study (2008–2013) was a £2.3 million research study funded by the UK Engineering and Physical Sciences Research Council. The core project team comprised nine investigators based in nine institutions around the UK, which included a range of disciplinary perspectives and expertise across the fields of transport, energy and carbon, environmental sciences, civil engineering, computer science, urban modelling, physical activity, public health, and health economics and transport [26].

iConnect is a good example of how public health can contribute alongside other disciplines to the generation of evidence to inform decision-making processes relating to physical activity and the built environment. In public health, evidence with internal and external validity is highly prized and this is achieved by understanding how an intervention achieves population impact in a particular context or setting. Observational epidemiology and feasibility studies are used to develop pilot interventions, which are then tested in stages before conducting a large randomised controlled trial of effectiveness. Other disciplines tend to focus on case studies as the main design for generating evidence for understanding ‘the real world’ and place the evidence of experts and consultants in a higher place in the evidence hierarchy than is customary in public health. This is particularly because of the secondary order of peer review evidence in the broad fields encompassing the built environment in determining what policies and programmes go forward in working cultures characterised by opinions, not least those of elected councillors.

The challenge of evaluating the impact of an environmental intervention on physical activity within a complex system was met through the development of the iConnect conceptual methodological approach or logic model [19]. This was informed by a realist approach to evaluation [27], which advocates developing theory or a conceptual framework to identify the mechanisms that underpin use of new walking and cycling infrastructure in different contexts [28]. Logic models are important tools in public health to ensure that the complex nature of the system in which the research takes place is considered and understood sufficiently, in particular, in terms of how a given intervention (such as changes to the environment) impacts on outcomes (for example, physical activity levels). The core methods and core survey Transport and Physical Activity Questionnaire from iConnect were derived from a logic model and were combined with detailed objective measures from subsets of the study panel cohorts at 1 and 2 years (*n* = 1465) [29, 30]. iConnect outputs have helped to understand the process of behavioural change regarding physical activity [31], informing decision makers in central government, local authorities, active travel users and academia through active engagement of these key stakeholders not only with the findings, but also the whole evidence generation process. Overall, the work of the iConnect Consortium and its collaborators, Sustrans and Living Streets, has demonstrated the potential of a supportive built environment and infrastructure to promote walking and cycling to achieve change in physical activity at a population level [32].

### Bristol Cycling City

In June 2008, Cycling England and the Department for Transport awarded the urban area of Greater Bristol £11.2 million to invest in the promotion and encouragement of cycling through better infrastructure, training and promotion. A Cycling City target was to double the number of cycle parking spaces from around 4000. This was achieved by the end of Cycling City. The Council has installed approximately 150 stands (300 spaces) since Cycling City so there are 9000 public spaces.

Cycling City demonstration status brought about some significant changes. Cycle use rose by about 40 % during this period (already on an increasing trend) but the intervention accelerated the background trend of increases in cycle use. In 2011 16,211 Bristol residents commuted by bicycle (94 % increase on 2001). The average rate of people cycling to work in 2007 was 6.7 %. Figures from 2010 showed that 9.8 % of people cycled to work, with the Ashley area of the city showing over one in four people cycling to work (26 %). The areas of Bishopston, Redland and Southville also showed around one in five people cycling to work. The Gloucester Road, one of the city’s major roads, had a 14.8 % modal share for cycling by 2011, up from 7.6 % in 2002 [33].

The programme was guided by evidence-based support from members of the Public Health’s Department’s Healthy Urban Team; an initiative to embed public health professionals within the built environment activities of the city council, to effect change through the promotion of evidence-based public health approaches. One activity was the provision of plain English summaries of peer-reviewed studies regarding the effectiveness of different cycling-related interventions. Importantly, this information was requested by the Cycling City Manager. Without the close working relationship between public health and colleagues in transport planning that had been cultivated through the establishment of the Healthy Urban Team, the evidence would have remained unknown to transport planners and engineers who rarely access peer-reviewed studies.

There were other factors that helped increase cycle use such as the lowering of speed limits in many streets from 30 mph (48 kph) to 20 mph (32 kph) between 2010 and 2015. This initiative originated in the public health team and was guided throughout by a public health practitioner advising as to the evidence of effectiveness and cost effectiveness. Public health input at the highest levels of the council ensured that the 20-mph initiative followed an evidence-based approach. This approach was extremely important in advocating for action and ensuring that the policy was introduced with full consideration of the possible consequences. The public health practitioner also initiated research into the evidence of effectiveness of a ‘safety in numbers’ effect (presence of significant volumes of cyclists in a geographical area), which suggested a decrease in the rate of cycle crashes when viewed against the total number of cyclists.

## Conclusions

This article has outlined how public health can contribute to generating evidence to inform decision-making processes for designing environments to promote physical activity. As noted, there are significant challenges to promoting an evidence-based approach in this area. These include the complex environments in which interventions operate, in addition to disciplinary differences in approaches to evidence generation and use. Public health as a profession needs to work more closely on a routine basis with the range of professions and organisations that have responsibility for designing environments for physical activity. Knowledge and skills transfer across professional divides offers the potential to promote evidence-based approaches among those who traditionally have not engaged with that agenda. The task of improving the quality and scope of evidence in this field is also challenging. Public health needs to learn lessons from examples of good practice, to consider how best to evaluate the impact of environmental interventions on physical activity levels within complex systems. Despite these challenges, the case studies presented here offer a snapshot of how problems can be overcome to promote evidence-based approaches to designing environments for physical activity.
